# Precision Public Health Campaign: Delivering Persuasive Messages to Relevant Segments Through Targeted Advertisements on Social Media

**DOI:** 10.2196/22313

**Published:** 2021-09-24

**Authors:** Jisun An, Haewoon Kwak, Hanya M Qureshi, Ingmar Weber

**Affiliations:** 1 School of Computing and Information Systems Singapore Management University Singapore Singapore; 2 Yale School of Medicine Yale University New Haven, CT United States; 3 Qatar Computing Research Institute Hamad Bin Khalifa University Doha Qatar

**Keywords:** precision public health, tailored health communication, social media advertising, Facebook advertising, public health campaigns, effectiveness of campaigns, public health, advertising

## Abstract

Although established marketing techniques have been applied to design more effective health campaigns, more often than not, the same message is broadcasted to large populations, irrespective of unique characteristics. As individual digital device use has increased, so have individual digital footprints, creating potential opportunities for targeted digital health interventions. We propose a novel precision public health campaign framework to structure and standardize the process of designing and delivering tailored health messages to target particular population segments using social media–targeted advertising tools. Our framework consists of five stages: defining a campaign goal, priority audience, and evaluation metrics; splitting the target audience into smaller segments; tailoring the message for each segment and conducting a pilot test; running the health campaign formally; and evaluating the performance of the campaigns. We have demonstrated how the framework works through 2 case studies. The precision public health campaign framework has the potential to support higher population uptake and engagement rates by encouraging a more standardized, concise, efficient, and targeted approach to public health campaign development.

## Introduction

**Figure 1:**
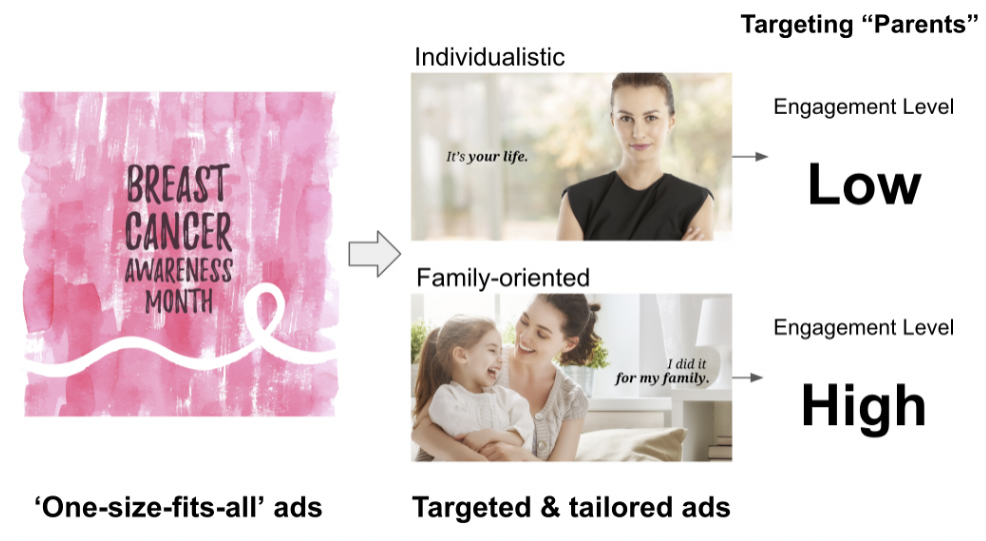
An example advertisement of precision public health campaign compared with a traditional one-size-fits-all advertisement.

In recent years, medicine has been transitioning from a homogeneous, *all-encompassing* approach to a vision of *precision medicine*, where each patient receives personalized treatment based on their respective genomics, demographics, lifestyle, and other factors. However, public health campaigns have largely remained one-size-fits-all. Although established marketing techniques, such as buzz marketing [1,2], branding [3,4], and social marketing [5-8], have been applied to design more effective health campaigns, often a uniform message is broadcasted to large populations, irrespective of their members’ unique characteristics (Figure 1). Arguably, the inflexibility of the traditional approach to public health campaigns [9] decreases campaign effectiveness by making the audience feel less engaged [10,11]. At the same time, previous studies have described low participation rates on questionnaires otherwise intended to effectively engage populations in designing tailored interventions [12], leaving a critical gap between public health needs and campaign success. In this regard, individual targeting may help create a bridge.

As individual digital device use has increased, so have individual digital footprints, creating potential opportunities for targeted digital health interventions [13]. The traces people leave online can be used to infer their personal preferences, political attitudes, physical activities, and psychological characteristics [13]. From a public health standpoint, these digital footprints may prove crucial for implementing more effective precision public health campaigns (PPHCs).

Beyond online search engine data, which are already being used to influence digital health interventions [14,15], the relevance of footprints captured by *likes*, *comments*, and *shares* on social media platforms, including Facebook, Twitter, and Instagram, remains largely unanalyzed and unexplored. Compared with traditional mass media channels, the targeted advertising tools (TATs) available through such sites are already being used by some researchers to recruit study participants [16], create representative samples [17], identify people with particular characteristics [18,19], and obtain public health insights in the United States [20]. Facebook advertising particularly has received increased attention and use in health communication research, especially for online recruitment, likely because of its diverse user base, broad reach, and cost-effectiveness [21-26]. One study used an 11-week Facebook advertising campaign to recruit a cohort of Michigan Facebook users aged 18-64 years [25]. The campaign reached 1.88 million users and only cost US $15,000.

Beyond reaching a wider audience, TATs such as Facebook advertising also offer a time and cost-effective methodology to identify and engage with specific smaller subsets of populations on precision public health. For example, Pedersen et al [24] recruited 1023 young adult veterans by targeting a population aged 18-40 years living in the United States with listed interests in veteran- or military-themed video games, such as the *Call of Duty* series. Each of their Facebook advertisements ran between US $0.33 and US $0.66 per click. Similarly, Reiter et al [26] used Facebook TATs to recruit young gay and bisexual men for a human papillomavirus (HPV) vaccination intervention. They first selected English-speaking males in the United States aged 18-25 years, then selected for anyone with listed interests in bisexuality; homosexuality; same-sex relationship; genderqueer; gay pride; lesbian, gay, bisexual, and transgender (LGBT) community; LGBT culture; or rainbow (LGBT movement). Their campaign reached 35,646 users at a total cost of US $413.72, with a cost per click (CPC) of US $0.58.

Ultimately, although previous research studies have used TATs to run public health campaigns, standard systematic evaluation metrics for public health campaign effectiveness and engagement are yet to be described. Consequently, in this study, we propose a novel PPHC framework to structure and standardize the process of designing and delivering tailored health messages to target particular population segments using social media TATs. Specifically, we outline five critical stages: (1) defining a campaign goal, priority audience, and evaluation metrics; (2) splitting the target audience into smaller segments; (3) tailoring the message for each segment and conducting a pilot test; (4) running the health campaign formally; and (5) evaluating the performance of the campaigns.

## Development of the Framework

On being tasked with designing a targeted advertisement to promote breast cancer screening in Qatar using Facebook and Instagram (see case study involving breast cancer screening below), we initially developed the PPHC framework as a means to systematically run a public health campaign on social media. This task was challenging because of the restrictions and limitations of TATs and the absence of an existing framework to provide step-by-step guidance on designing and implementing public health campaigns using TATs. The first 3 stages of the framework were developed alongside the process of understanding the functionalities and limitations of the TAT. These steps were refined based on experiences from designing a separate Qatar flu shot campaign case study. The final framework outlined below was then expanded to include postcampaign data analysis evaluating online and offline impact, with the impact being defined as higher click-through rates (CTRs).

## Description of the PPHC Framework

### Overview

**Figure 2:**
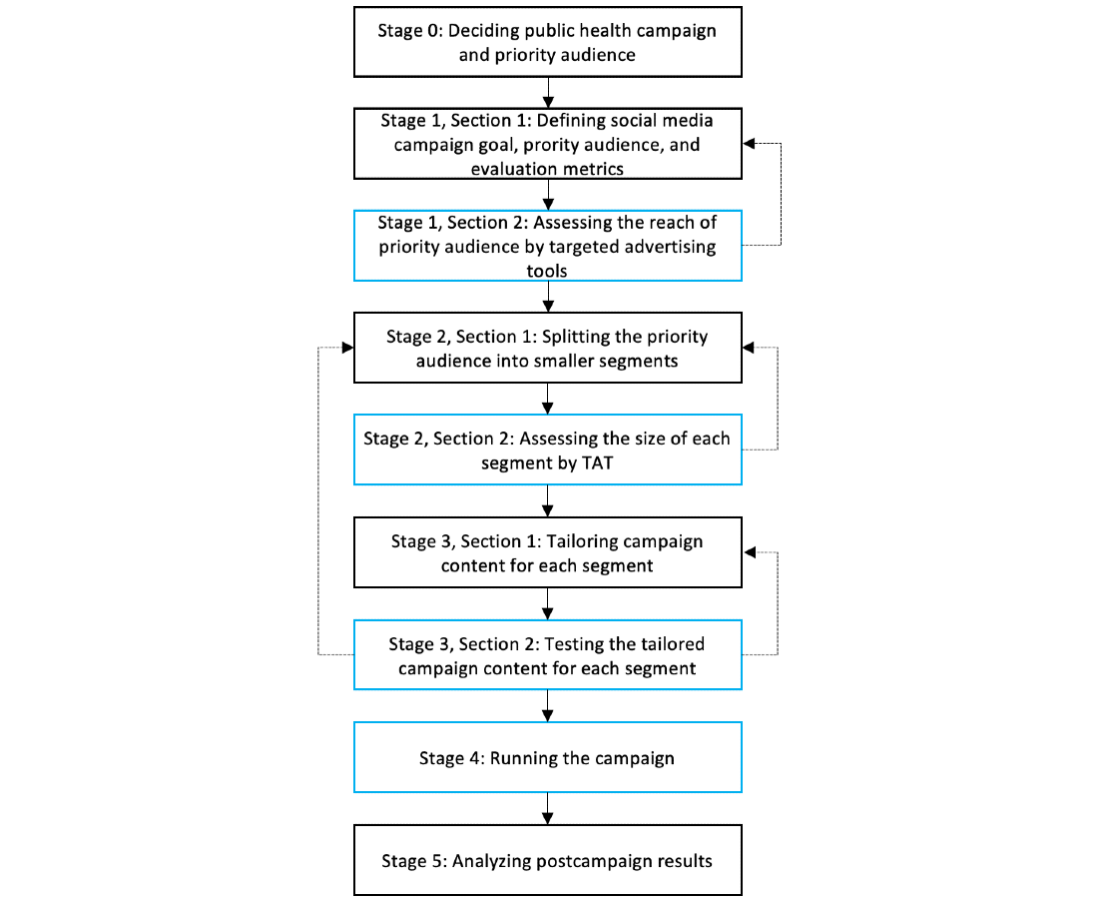
Stages of the precision public health campaigns framework. Dotted lines indicate optional paths to revisit if necessary. The blue boxes indicate a stage that uses targeted advertising tools. TAT: targeted advertising tool.

We define the PPHC framework as consisting of five stages (Figure 2): (1) defining campaign goal, priority audience, and evaluation metrics (eg, we aimed to promote breast cancer screening among women in Qatar aged ≥45 years using Facebook and Instagram, and the performance is measured by CTR); (2) splitting the target audience into smaller segments (eg, we targeted the Arab group and Filipino group); (3) tailoring the message for each segment and doing a pilot test (eg, we used the same message [“I did it for myself”] but used culturally resonant models for the advertisement image); (4) running the health campaign formally; and (5) evaluating the performance of the campaigns (eg, we examined whether culturally resonant advertisements would have higher CTRs). Stages 1-3 can be further subdivided into 2 iterative sections, where the results of the second section can tentatively validate the decisions made in the first section using TATs.

The PPHC framework has the following benefits in different stages of running the public campaigns:

1. Before running the campaign
  Estimating the size of target audience segments
  Quick and cheap pilot testing
2. Running the campaign
  Accurate targeting
  Real-time tracking of the reach
3. After running the campaign
  Assess the effectiveness of campaigns

### Stage 0: Deciding Public Health Campaign and Priority Audience

Before applying the PPHC framework, we assume that researchers and practitioners have already determined their public health campaign’s goals and defined their target demographic group using prior literature in consultation with experts in the field.

### Stage 1: Defining Social Media Campaign Goal, Evaluation Metrics, and Target Audience

Stage 1 consists of 2 iterative stages. In stage 1, section 1, social media campaign goal and evaluation metrics are set. In stage 1, section 2, the reachability of the target audience can be assessed using TATs. Most social media giants, including Facebook and Instagram, Twitter, LinkedIn, Snapchat, and TikTok, generate revenue from advertisements [27]. These platforms allow users to create detailed profiles of their users, including demographic attributes, such as age, gender, spoken language, living location, income range, and political leaning. Using built-in TATs, advertisers can explore these attributes to identify targeting criteria for their advertisements. TATs can then provide advertisers with audience reach estimates based on their selected criteria. For example, the estimated number of Facebook users who are female, aged 20 years, very liberal, and interested in *The New York Times* is 160,000. Facebook advertisement audience reach estimates have already been used as a proxy to measure the scope of various online populations across multiple domains [28-30], with many other social media platforms offering similar estimation features. Thus, the proposed PPHC framework uses audience estimates to check for the reachability of the target audience. If the target audience cannot be reached, return to stage 1, section 1, and iteratively revise it.

### Stage 1, Section 1: Defining Social Media Campaign Goal, Target Audience, and Evaluation Metrics

The first stage of the PPHC framework defines the campaign goal, target audience, and evaluation metrics for social media campaigns. For instance, a ministry of health running health campaigns on obesity defined their priority audience at stage 0, as all parents with children aged <18 years may define their social media campaign goal at stage 1 to be *raising awareness for childhood obesity* or *increasing children’s obesity clinic registration rates*. At stage 1, we initially consider the priority audience and target audience to be the same.

Next, to define the evaluation metrics, several considerations must be considered. First, the metrics should be quantitatively measured. In the example of raising awareness of childhood obesity, awareness itself is not directly measurable. Thus, a proxy to reflect the level of awareness should be designed. Such proxy measures can include survey results (pre- and postcampaign), corroboration of trends by literature review, or other record analysis. Second, the evaluation metric must be measured online or offline. Online metrics are typically easier to measure than offline metrics. Third, if the campaign calls for any kind of action, the metric must be quantifiable by measuring the frequency of that action (eg, number of visits to the website, number of cancer screening registrations, and number of vaccination shots) after the campaign intervention. Finally, in some cases, it may be helpful to create a *dedicated endpoint* to collect the data of the target audience (ie, those who are exposed to social media campaigns). For example, a dedicated website (or additional parameter in the URL to mark visits by the target audience), a newly created contact email address, or telephone number can be used to mark visits by the target audience from all other visits. In a broad sense, if an individual who is exposed to the campaign can be identified through a coupon code or additional survey (eg, asking for reasons to visit) at the offsite (eg, clinic), it falls in this case.

When the campaign uses offline metrics for evaluation, there is one additional consideration—whether the campaign will use randomized controlled trials (RCTs). Although not all TATs support RCTs, it is possible to divide a control group and a treatment group online using TATs [31]. In this case, the impact of the campaign can be estimated more accurately by controlling for other confounding factors. We explain how to implement an RCT through TATs in stage 2, but the PPHC framework recommends making a decision on whether to adopt an RCT in stage 1.

To find engaging content for a target group, Facebook TAT also supports an automated service called *dynamic creative*, which integrates multiple advertisement components (eg, images, videos, titles, descriptions, and call-to-action buttons) to improve advertisement delivery via optimization [32]. Dynamic creative automatically selects which creative variations to show each member of the target group based on their unique subcharacteristics to maximize advertisement impact (eg, number of clicks).

If one aims to reach the largest number of people via advertisement clicks, using dynamic creative is an option. However, it is worth noting that the tool optimizes advertisement exposure to reach a higher CTR potentially at the expense of including others from diverse backgrounds, as the algorithm may begin showing the advertisements to only those users who are more likely to click on the advertisement in the first place. Consequently, dynamic creative might not be a beneficial tool when designing a public health campaign.

1. Campaign goal: What should be achieved through the health campaign?
2. Target audience: Who is the health campaign intended to target?
3. Evaluation metrics:
  Online metrics: for example, number of clicks, number of downloads, visits to websites, survey results, etc.
  Offline metrics: for example, number of visits to clinics, etc.
4. Evaluation plan:
  Dedicated endpoints
  RCTs

### Stage 1, Section 2: Assessing the Reach to the Priority Audience by TATs

#### Overview

Although social media use has witnessed an uptick in recent years, some populations, including older adults, remain underrepresented. Thus, before continuing campaign design, it is important to check whether social media channels are an appropriate medium through which to reach the priority audience. TATs allow for audience size estimation using a diverse set of traits as inclusion criteria. In general, criteria can be grouped into four categories: location, demographic, behavioral, and interests. In the following sections, we describe the demographic traits and locations available on the Facebook advertising platform. We have described the other categories in stage 2, section 2. The full list of subcategories can be found in Multimedia Appendix 1. We noted that criteria availability may vary across different social media platforms and regions (eg, Facebook supports income level for its US users only).

#### Demographic Traits

Demographic traits–based targeting is a shared feature of TATs across most social media services. Gender-, age-, and language-based targeting is commonly supported. We noted that Facebook categorizes *race* as a behavioral trait rather than a demographic trait:

- Gender (all, men, and women)
- Age (13 to ≥65 years)
- Education (eg, education level and fields of study)
- Financial (eg, household income)
- Life events (eg, anniversary, away from family, date of birth [month of birth and upcoming birthday], and new job)
- Parents (eg, with toddlers and preschoolers)
- Relationships (eg, single, in a relationship, married, and divorced)
- Work (eg, employers, industries, and job titles)

#### Locations

TAT in most of the social media services offers a location-based targeting. Using locations, it is possible to target worldwide (eg, type “worldwide”), by country group or geographic region (eg, type in “Asia”), by subregions within a country (eg, type in “Michigan”), by free trade area (eg, type in “GCC” or “Gulf Cooperation Council”), or by other features (eg, type in “iTunes app store countries” or “emerging markets”).

Alternatively, it is also possible to manually select a certain area. For example, one can set a manual area with a radius of 5 km from a given point on the map. TATs will then only be used to provide an audience reach estimate and target people who live, commute, or work in that area and use the platform.

**Figure 3:**
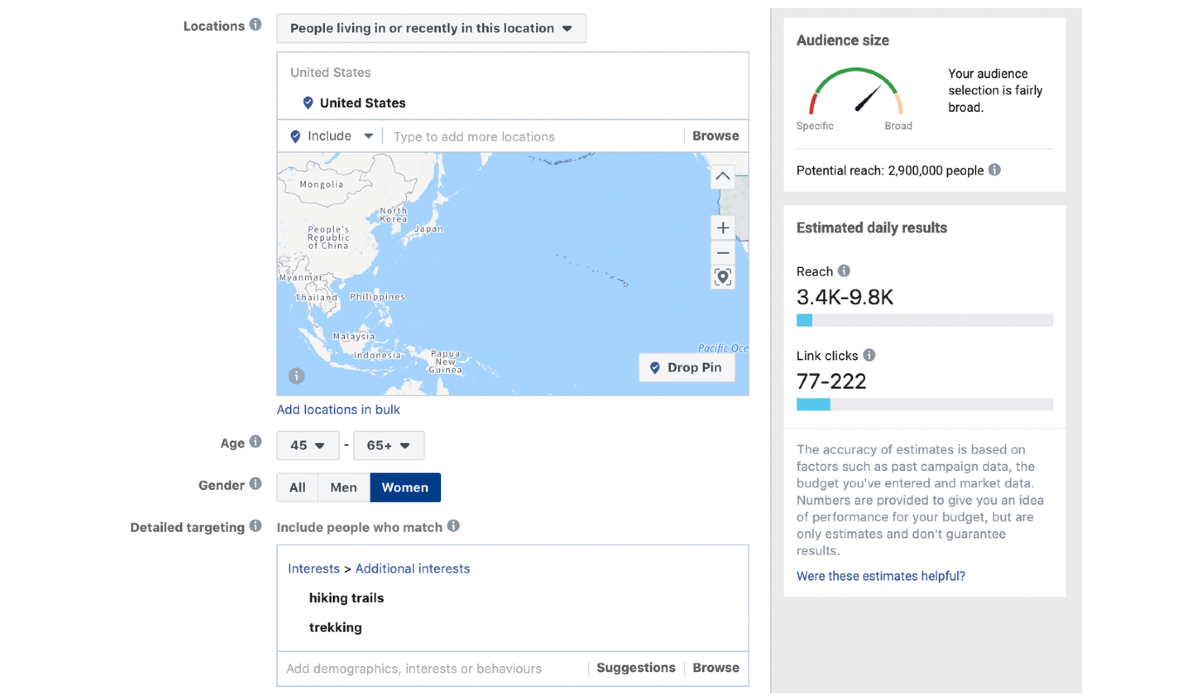
Facebook advertising tool interface. The potential reach on the right side bar shows the approximate audience size of targeted users.

Figure 3 shows interfaces of TATs on Facebook and Instagram as an example. Since Instagram was purchased by Facebook on April 9, 2012, users on either of the two social media platforms can be targeted via Facebook TATs. The tool shows that the approximate audience size (potential reach on the right side bar) is 2.9 million (including both Facebook and Instagram accounts) when targeting users who are women, living in or recently in the United States, aged ≥45 years, and interested in trekking and hiking trails.

Although existing social media advertising platforms largely share their interfaces when it comes to targeting, the attributes available for targeting vary widely across platforms. Table 1 compares which demographic and location traits are available on the 4 most popular social media platforms, namely, Facebook and Instagram, Twitter, Snapchat, and TikTok, in the United States, all of which support basic demographic traits, such as age, gender, and language. However, rich demographic traits are only available on Facebook and Snapchat and not on Twitter or TikTok. Regarding location, all 4 platforms support country-, state-, or region-level location-based targeting. City- or zip code–level targeting can also be used on all platforms except TikTok.

**Table 1:** Available demographic and location traits for targeting in various social media platforms.

			Facebook and Instagram		Twitter		Snapchat		TikTok
**Demographic traits**									
	Age (years)	Yes		Yes (by range)		Yes		Yes (by range)	
	Gender	Yes		Yes		Yes		Yes	
	Language	Yes		Yes		Yes		Yes	
	Financial	Yes		No		Yes		No	
	Political leaning	Yes		No		No		No	
	Education level	Yes		No		Yes		No	
	Marital status	Yes		No		Yes		No	
	Parents	Yes		No		Yes		No	
	Occupation	Yes		No		Yes		No	
	Parents	Yes		No		Yes		No	
	Life events	Yes		No		Yes		No	
	Birth month	Yes		No		No		No	
**Location traits**									
	Worldwide	Yes		Yes		Yes		No	
	Country	Yes		Yes		Yes		Yes	
	State or county	Yes		Yes		Yes		Yes	
	City	Yes		Yes		Yes (DMA^a^)		No	
	Drop pin+1-80 km	Yes		No		No		No	
	Zip or postal code	Yes		Yes		Yes		No	

^a^DMA: Designated Market Area.

#### Adjustments

##### What Happens If TATs Do Not Have Enough Traits to Target the Priority Audience Defined in Stage 1, Section 1?

Although TATs offer a wide range of traits, some members of the priority audience might be missed when selecting for those traits. For example, assume that one wants to run a public health campaign for screening hypertension. As it is known that high blood pressure can run in families, the priority audience might be defined as those with a family history of hypertension. However, it is impossible to target this population on social media using TATs because such sensitive information is neither accessible nor available on social media via TATs. Similarly, TATs do not allow advertisers to target people based on their nationality because of potential misuse for discrimination. In some cases, other traits may be used as proxies. For example, language might serve as a proxy for nationality in particular countries (eg, Korean-Korea). If no proxies exist, delivering targeted health campaigns for the desired priority audience may be difficult. When this is the case, consider the feasibility of using a broader audience in stage 1, section 1.

##### What Happens If the Target Audience Is Too Small?

If the estimated size of the priority audience is too small, the targeting criteria should be carefully examined to determine whether it is too strict. If it is possible to loosen some conditions, do so and recheck the estimated reach again until the estimated audience size is sufficient. In addition, for some target groups, it is possible that the corresponding social media service is not an appropriate channel at all. For example, older adults (aged >65 years) rarely use Facebook. Those people will be better targeted by other approaches, such as offline campaigns through community centers.

### Stage 2: Audience Segmentation

In stage 2, the aim is audience segmentation. This stage consists of 2 sections. In stage 2, section 1, the priority audience is split into smaller segments. In stage 2, section 2, the reachability of each segment is assessed by TATs. If a certain segment cannot be reached, return to stage 2, section 1 and adjust the segmentation.

### Stage 2, Section 1: Splitting the Priority Audience Into Smaller Segments

Once one social media service has been confirmed to be the appropriate platform to reach the priority audience (stage 1), the traditional one-size-fits-all style campaign can definitely be run on that social media. However, for an efficient PPHC, audience segmentation is a natural next step [33,34].

Audience segmentation is the process that divides the audience (sometimes a population or market) into smaller groups whose members share unique properties [34]. Audience segmentation in health campaign domains is well reviewed in a study by Slater [35].

Segmentation can be performed using various demographic traits. For instance, one can design different advertisements for each gender or for persons of different age groups. Beyond these simple divisions, researchers in marketing domains have found that persuasive appeals are more effective in influencing behavior when they are tailored to individuals’ unique psychological characteristics [36]. For example, people who are extroverted might react differently to a given campaign stimulus than people who are introverted. Thus, one could envision using such personality or psychological dimensions for audience segmentation purposes.

If a team decided to implement RCTs in stage 1, they should examine whether it would be possible to define a control and treatment group for an online controlled experiment [31] on the given social media service. Not all TATs are equipped to run RCTs. On Facebook, one can set up an environment for conducting RCTs by splitting the Facebook population into two random groups (RGs) with one of the Facebook targeting criteria, birth month. This results in having two RGs: people whose birthdays are in odd months (odd-month group) and those whose birthdays are in even months (even-month group). As these 2 groups are mutually exclusive and birth month is unlikely to be correlated with health behaviors or demographics, 1 group can be considered the control group and the other can be considered the treatment group. The ensuing campaign can be designed to expose targeted advertisements to the treatment group alone, excluding the artificial control group. In doing so, one can obtain offline data such as clinic visits aggregated by birth month, enabling impact measurement with minimal privacy risk. For example, if the campaign is on flu vaccination rates, then campaigns through TATs could target only those born in odd months (or even months). A comparison of the number of those who got flu shots between the control and treatment groups would show the impact of the campaign. However, there can be spillover effects, as a person who has received a message (treatment group) might reshare that message with a person who has not (control group), thereby leading to an underestimation of campaign impact. This risk should be fully considered, especially when running an RCT for an extended period.

An RCT can be conducted with any trait that can split a target population into 2 or more RGs. For example, birth year attributes can be used instead of birth month attributes. In this case, people born in odd years (odd-year group) can be considered as a control group and people who are born in even years (even-year group) can be considered as a treatment group. Geographic splitting is another option. Here, the city or postal code attributes can be used to define RGs, such as odd and even zip codes. As shown in Table 1, birth month attributes are only available on Facebook and not on other platforms. However, Snapchat has birth year attributes, and Twitter can use geographic splitting to run an RCT.

Beyond using the traits of TAT, an RCT can be designed using a custom audience. On TAT, a custom audience is a type of audience created from a customer list (such as email address, phone number, and address). A TAT matches existing customer information with social media users, thus allowing a campaigner to run a targeted campaign using matched users. A campaigner can then split users into the customer list to gain 2 random custom audience groups. All 4 social media platforms support targeting custom audiences.

### Stage 2, Section 2: Assessing the Size of Each Segment by TAT

#### Overview

After deciding which segmentation to follow, the next step is to assess whether the segmentation is possible and has enough reach through social media. Beyond the targeting criteria introduced in stage 1 (ie, demographic traits and locations), TATs offer an extensive set of behavioral and interest traits that can all be used for targeting. In the following sections, we describe these 2 categories in more detail with example traits.

#### Behavioral Traits

Different social media services provide different levels of audience targeting based on behavioral traits. For example, Facebook allows for audience targeting by combining various features, such as which country a user used to live in, which type of device they use to connect to Facebook, or whether they are frequent travelers. Some of these targeting options are not available on Twitter, Snapchat, or TikTok. Examples of behavioral traits provided by Facebook are as follows:

- Consumer classification (eg, people who prefer high-value goods)
- Digital activities (eg, console gamers, early technology adopters, and small business owners)
- Expats (eg, lived in a certain country or lives abroad)
- Multicultural affinity (eg, African American [United States], Asian American [United States], and Hispanic [United States-all])
- Purchase behavior (eg, engaged shoppers)
- Travel (eg, commuters and frequent international travelers)

#### Interests Traits

These traits are mainly divided into 9 categories, 4 of which are listed below:

- Family and relationships (eg, dating and parenting)
- Fitness and wellness (eg, meditation, physical exercise, running, weight training, and yoga)
- Hobbies and activities (eg, home and garden and travel)
- Additional interests (eg, breast cancer awareness, herbal tea, and National Vaccine Information Center)

All these traits can be used for audience segmentation in the PPHC framework. For better audience segmentation, the target traits should be shared within each segment but not across the segments. Generally, a combination of different variables is recommended for better audience segmentation [37,38]. We note that some of the behavioral traits are not available on other platforms. For example, attributes such as expats (lived in a certain country), multicultural affinity, politics, Ramadan, and frequent travelers are only available on Facebook as a targeting feature. However, we report that the other 3 platforms offer extensive and fine-grained interest traits that include most of Facebook’s attributes. The full list of attributes the four social media platforms offer can also be found in Multimedia Appendix 1.

If one wants to segment their audience by personality for the campaign, they can use a set of interest traits to target people who are extroverted or introverted. Recent research shows that people’s psychological characteristics can be accurately predicted from their digital footprints, such as Facebook likes or tweets [36].

For example, a recent study showed that the list of introverted target likes included *Stargate* and *Computers*, whereas the list of extroverted target likes contained *Making People Laugh* or *Slightly Stoopid*.

Then, as in stage 1, section 2, TAT measures the size of each audience segment on social media. If the size of any segment is not sufficient to run, audience segmentation should be refined.

### Stage 3: Tailoring the Campaign Content

In stage 3, the goal is to tailor the campaign content for each segment determined in stage 2. In stage 3, section 1, candidate campaign content is created. Then, in stage 3, section 2, content for each segment is tested. If the testing result is not satisfied, return to stage 3, section 1 and revise the content.

### Stage 3, Section 1: Tailoring Content for Each Segment

In this stage, the actual health campaign content is created for each audience segment. The campaign contents, including messages and pictures, need to be carefully designed to maximize their appeal for each of their respective audience segments. Scholars in the marketing field have extensively studied differences in consumer behavior across gender, age, location, and culture [39], all of which can offer valuable insights to campaigners.

### Stage 3, Section 2: Testing the Tailored Messages for Each Segment

Once the campaign contents are prepared, the campaigner can test whether they are well tailored for each of the audience segments by running the campaign through TAT on a small scale. TAT typically allows individuals to run campaigns on a fairly low-budget sample limit. On Facebook, the minimum budget is US $1 for running a campaign.

There are a wide variety of measures that can test the effectiveness of tailored content, including CTR; CPC; number of website visits; and number of shares, likes, or comments.

A simple CTR can be used as a measure to test whether a given campaign holds an appeal with the targeted audience segment. The small-scale pilot test follows the form of A/B testing. By comparing the CTR between the segments and the campaigns, it can be determined which campaign performs the best for each segment. We note that the A/B testing feature is available on other platforms such as Snapchat and TikTok, except Twitter. With the A/B testing feature, it would be clearer and easier to create A/B testing. However, the absence of the A/B testing feature would not stop performing A/B testing on Twitter. One can simply create two advertisement sets with different content but with the same audience.

For example, when a campaigner creates content A for the audience segment A and content B for segment B, content effectiveness can be assessed by running four campaigns where all possible combinations of content and population segments are tested: content A and segment A, content B and segment A, content A and segment B, and content B and segment B. The results of the 4 campaigns, measured by the CTR, can suggest whether the campaign content is well suited for the targeted audience segment. If the CTR of content A and segment A is higher than that of content A and segment B and that of content B and segment A, it means that content A is better for segment A.

When no significant differences are found, either (1) enhance the campaign contents or (2) go to stage 2, section 1 and split the priority audience in a different way.

### Stage 4: Run the Campaign

Once tailored health campaigns for each user segment are confirmed through pilot tests, they are ready to run the health campaign formally. TAT provides real-time tracking of the performance of health campaigns, such as reach, CTR, and consumed budgets.

As stated in stage 1, evaluation plans are carefully considered when formally running the campaigns. For example, if a control and treatment group split by birth month is required for evaluation, each audience segment is divided into those who were born in the odd month and those who were born in the even month and set control and treatment groups, and the campaign will be run for treatment groups.

### Stage 5: Analyzing Postcampaign Results

The final stage of the framework is the evaluation of the postcampaign results. As explained in stage 1, the evaluation aims to measure the impact of health campaigns. When dedicated endpoints are prepared, the impact of health campaigns can be measured directly using access data to those endpoints (eg, how many people make reservations via the dedicated website). When control and treatment groups are prepared, the difference in the CTR between the two represents the health campaign impact.

## Application of PPHC Framework: Two Case Studies

The following section provides researchers with practical examples of how the framework can be applied for running public health campaigns on social media (eg, Facebook).

### Case Study 1: Public Health Campaigns for Breast Cancer Screening in Qatar

#### Overview

To demonstrate the concept of PPHCs, we ran a small-scale Facebook advertising campaign to test culturally resonant advertisements in promoting breast cancer screening and flu vaccination in Qatar under the PPHC framework.

On consultation with the Qatar Biomedical Research Institute, Hamad Bin Khalifa University Institutional Review Board, our case studies were deemed exempt from institutional review board oversight for human research participant protection.

#### Stage 0: Deciding Public Health Campaign and Priority Audience

We first define the goal and the target demographic group of our campaign about breast cancer screening in Qatar:

1. Campaign goal: the goal of the campaigns was to raise awareness of breast cancer screening in Qatar.
2. Priority audience: the American Cancer Society and Qatar Cancer Society recommend annual screening mammography for women aged >45 years [40]. Following the recommendation, we targeted women living in Qatar aged ≥45 years.

#### Stage 1, Section 1: Defining Social Media Campaign Goal, Evaluation Metrics, and Target Audience

We set the goal, target audience, and evaluation metrics for social media campaign. The campaign goal and target audience can be adjusted based on what the TAT offers:

1. Social media campaign goal: the goal of this campaign was to measure the effectiveness of culturally resonant advertisements in promoting breast cancer screening on social media.
2. Target audience: at this stage, we assumed that our target audience was the same as the priority audience. Thus, our target audience was women living in Qatar aged ≥45 years.
3. Evaluation metrics: we used the CTR (the proportion of the number of clicks by the total number of impressions) as our metric to evaluate the performance of the campaigns, in particular its resonance.

#### Stage 1, Section 2: Assessing the Reach to the Priority Audience by TATs

Our priority audience was women aged ≥45 years living in Qatar. On Facebook TAT, we set the audience by choosing the following three attributes: (1) location is Qatar, (2) gender is female, and (3) age is ≥45 years. The TAT estimated the potential audience reach (the number of users who satisfy the selected conditions) to be 66,000 on Facebook. As the reach was large enough to run the campaign, we were able to move to the next stage.

#### Stage 2, Section 1: Splitting the Target Audience Into Smaller Segments

To measure the effectiveness of culturally resonant advertisements, we further defined two subtarget groups: (1) the Arab group and (2) the Filipino group.

#### Stage 2, Section 2: Assessing the Size of Each Segment by TAT

To target the Arab group, we added one additional targeting criteria, *Arabic speaking*, given the base group (18,000). To target the Filipino group, we added *Lived in Philippines* to the targeting criteria (13,000). As each group was large enough, there was no need to revise the segmentation and moved to the next stage.

#### Stage 3, Section 1: Tailoring Campaign Content

As a base template, the advertisement image contained one female model, confident facing front on the right side, with a message, “It’s your life,” in two languages: the native language of the subtarget group (Arabic or Filipino) and English on the left side of the advertisement image.

**Figure 4:**
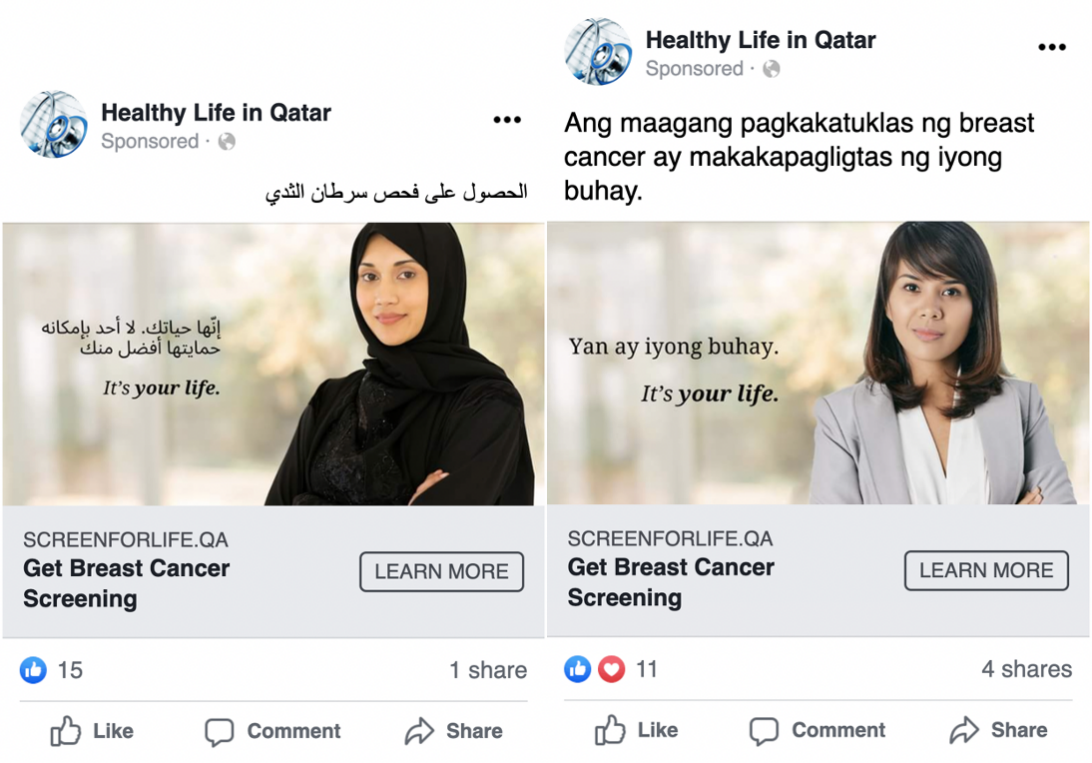
Culturally resonant advertisements for promoting breast cancer screening for Arabs (left) and Filipinos (right).

Then, we created two culturally specific advertisements—one advertisement image had an Arab (single woman) model (Figure 4, left), whereas another advertisement image had a Filipino model (Figure 4, right). We used the same background and font as the texts on the 2 advertisements. In addition, both advertisements had the same English headlines (“Get Breast Cancer Screening”) and main text (“Early detection saves your life”) in the corresponding language.

#### Stage 3, Section 2: Testing the Tailored Content for Each Segment

We ran a 3-day Facebook advertising campaign targeting the two subgroups described in stage 2 about breast cancer screening, which cost US $120. We used a split test, a random A/B testing function provided by Facebook. Given a target group (Arab group or Filipino group), Facebook randomly split the group into 2 groups and exposed 1 advertisement to each group. Thus, we were able to examine which advertisement was more attractive to the target group.

The campaign reached 17,734 Qatar Facebook users, yielding 392 website clicks across four advertisement sets. Table 2 shows the number of clicks, number of people who saw the advertisement, CTR, and CPC (US $). For example, for the Arab group, the Arab model advertisement yielded 129 clicks among 4636 people, resulting in a CTR of 2.78% and US $0.2 CPC. Our experiment showed that culturally resonant advertisements increase CTRs. The chi-square test with Yates continuity correction revealed that the CTRs significantly differed between culturally and nonculturally resonant advertisements (N=8867; *X*^2^_1_=15.9; *P*<.001; φ=0.04; odds ratio 1.83, 95% CI 1.36-2.45). For the Arab group, the advertisement with the Arab model resulted in CTRs of 2.78% (129/4636), an increase by a factor of 2 compared with the advertisement with the Filipino model (CTR: 63/4771, 1.32%). We found a similar trend among the Filipino group, with an increased CTR by a factor of 1.5.

On average, the culturally resonant advertisements resulted in a CTR of 2.85% (SD 0.07), which is higher than the average CTR of Facebook across all industries, which is 0.89% [41]. Although there might be cultural factors influencing CTRs among Filipinos and Arabs engaging with advertisements in general, our high CTR reinforces the potential gain of running targeted public health campaigns.

**Table 2:** Summary of the results of cultural targeting.

Advertisement	Arab group					Filipino group			
	Participants, n	Clicks	Click-through rate (%)	Rate per click (US $)	Participants, n		Clicks	Click-through rate (%)	Rate per click (US $)
Advertisement for Arab	4636	129	2.78	0.20	4151		78	1.88	0.38
Advertisement for Filipino	4771	63	1.32	0.47	4176		122	2.92	0.24

#### Stages 4 and 5: Running the Campaign and Evaluating the Performance of the Campaigns

As the purpose of this case study was to demonstrate how the PPHC framework could be applied in a real-world example, we omitted to proceed to stages 4 and 5. We further discuss how our framework can be used to evaluate the effectiveness of public health campaigns by measuring changes in offline behavior.

### Case Study 2: Public Health Campaigns for Promoting Flu Vaccination in Qatar

The second case study is for promoting flu vaccination in Qatar.

#### Stage 0: Deciding Public Health Campaign and Priority Audience

We first define the goal and the target demographic group of our campaign on flu vaccination in Qatar:

1. Campaign goal: the goal of the campaigns was to raise awareness and increase the uptake of flu vaccination in Qatar.
2. Priority audience: according to the Centers for Disease Control Prevention, “all persons aged 6 months of age and older are recommended for annual vaccination, with rare exception” [42]. Following this recommendation, we targeted everyone living in Qatar.

#### Stage 1, Section 1: Defining Campaign Goal, Priority Audience, and Evaluation Metrics

We set the goal, target audience, and evaluation metrics for social media campaign. The campaign goal and target audience can be adjusted based on what the TAT offers:

1. Social media campaign goal: the goal of this project was to measure the effectiveness of gender in promoting flu vaccination in Qatar on social media.
2. Priority audience: following the rule of online advertising restriction to children and young people aged <18 years, we targeted everyone living in Qatar aged ≥18 years.
3. Evaluation metrics: we used the CTR (the proportion of the number of clicks by the total number of impressions) as our metric to evaluate the performance of the campaigns.

#### Stage 1, Section 2: Assessing the Reach to the Priority Audience by TATs

Our priority audience was people living in Qatar aged ≥18 years. On Facebook, the number of users who match these conditions was 2.4 million.

#### Stage 2, Section 1: Splitting the Target Audience Into Smaller Segments

To measure the effect of gendered advertisements for promoting flu vaccination, we further defined two target subgroups: female and male.

#### Stage 2, Section 2: Assessing the Size of Each Segment by TAT

We targeted the female and male groups by adding one additional targeting criterion, *gender*. The estimated sizes of the female and male groups were 550,000 and 1.8 million on Facebook, respectively. As each group was large enough, there was no need to revise the segmentation, and we moved on to the next stage.

#### Stage 3, Section 1: Tailoring the Message for Each Segment and Doing a Pilot Test

**Figure 5:**
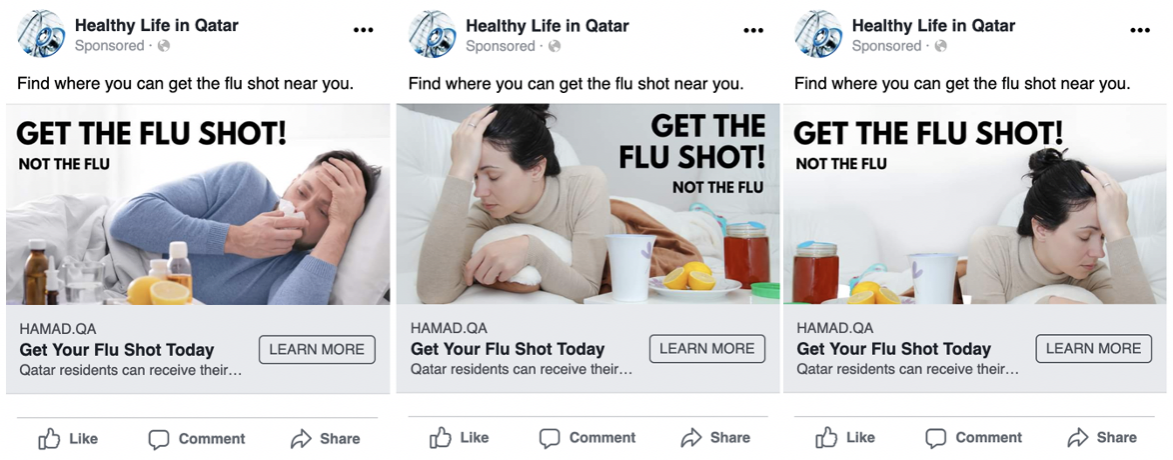
Gender-based advertisements for promoting flu vaccination (left: male model, center: female model [original], and right: female model [mirrored]).

We created 2 gendered advertisements. One advertisement image had a single female model (Figure 5, center), whereas the other had a single male model (Figure 5, left). In both images, the models are in the bed with their hands on their forehead, and both advertisements had the same headlines (Get Your Flu Shot Today), main text (Find where you can get the flu shot near you), and messages in the advertisement image (“GET THE FLU SHOT! NOT THE FLU”).

#### Stage 3, Section 2: Testing the Tailored Content for Each Segment

We designed a 3-day Facebook advertising campaign targeting the 2 male and female groups for flu vaccination. We again used a split test and measured which of the 2 contents has higher CTRs for each of the 2 groups.

After a half day of the experiment, we noticed that the female group had higher CTR for the advertisement with a male model (68/2830, 2.4%) compared with that with a female model (33/2742, 1.2%). Such a difference was not observed in the male group. The CTRs were 1.41% (53/3765) and 1.7% (68/3991) for the advertisement with a female and male model, respectively. Such results might have occurred because of the differences in the compositions of the 2 advertisement images. Compared with the advertisement with the male model, the advertisement with the female model has a darker background, and the text in the advertisement image was in two lines. To make the 2 advertisements more comparable, we created another advertisement content by mirroring the female model, brightening the background color, and positioning the texts in the same manner as in the advertisement with the male model. On creation of the new advertisement image, we ran a 3-day Facebook advertising campaign once again with all 3 advertisement content. As we had two subgroups with three advertisement content, with a US $10 daily budget, we spent US $180.

This campaign reached 109,983 Facebook users in Qatar in total, yielding 1830 website clicks across the six advertisement sets. Table 3 shows the number of clicks, number of people who saw the advertisement, CTRs, and CPC (US $). For example, for the female group, the female model advertisement (original) yielded 243 clicks among 15,347 people, resulting in a CTR of 1.58% and US $0.11 per click. Interestingly, we found that the mirrored version of the advertisement with the same female model had a slightly higher engagement with a CTR of 1.97% (301/15,300). The results indicate that the color and composition of advertisements also play an important role in user engagement. Surprisingly, the most engaging advertisement for the female group was the male model advertisement. The advertisement resulted in 390 clicks out of 16,572 people with a CTR of 2.35% and a CPC of US $0.07. This is a 48.7% increase in the number of clicks compared with the female model advertisement (original) and a 19.3% increase compared with the female model advertisement (mirrored). The difference in CTR between the female model advertisement (mirrored) and male model advertisement was statistically significant. The chi-square test with Yates continuity correction showed that the CTRs significantly differed between female (mirrored) and male advertisements (N=31,872; *X*^2^_1_=5.4; *P*=.02; φ=0.01; odds ratio 0.83, 95% CI 0.72-0.97).

**Table 3:** Summary of results of gendered targeting.

Advertisement	Female group			Male group	
	Total, N	Participants, n (%)	Total, N		Participants, n (%)
Advertisement with a female model (original)	15,347	243 (1.58)	19,171		264 (1.38)
Advertisement with a female model (mirrored)	15,300	301 (1.97)	19,380		293 (1.51)
Advertisement with a male model	16,572	390 (2.35)	22,960		339 (1.48)

For the male group, we found that advertisement content did not affect the level of engagement, whereas the female model advertisement (original) performed slightly less (CTR: 264/19,171, 1.38%) than the female model advertisement (mirrored; CTR: 293/19,380, 1.51%) and the male model advertisement (CTR: 339/22,960, 1.48%). However, these differences were not statistically significant.

#### Stages 4 and 5: Running the Campaign and Evaluating the Performance of the Campaigns

As the purpose of this case study was to demonstrate how the PPHC framework can be applied in a real-world example, we omitted to proceed to stages 4 and 5.

### PPHC for Evaluating Offline Impact

Thus far, we have demonstrated that a PPHC through social media advertising platforms is effective in terms of CTR. Here, we further discuss how it can be used to evaluate the effectiveness of public health campaigns by measuring offline behavioral changes.

As mentioned in *stage 1, section 1*, by splitting users into RGs, it is possible to conduct RCTs to measure offline behavior changes. Using Facebook’s TATs, we can use users’ birth months for random split, for example, let us assume we are running a campaign to promote breast cancer screening, we aim to evaluate whether cultural advertisements are more effective, and the evaluation metric is the number of people who visited the clinics for the examination. We also assume that aggregated, anonymous data on clinic visits by birth month, nationality, and other demographic attributes are ready and accessible.

To conduct an RCT, we first split users into 3 RGs using birth month attributes. Users born in January, April, July, or October are RG1 (1 mod 3), users born in February, May, August, or November are RG2 (2 mod 3), and users born in March, June, September, or December are RG3 (0 mod 3). We then considered RG1 as a control group. We did not show any advertisements to users in RG1. RG2 and RG3 are our treatment groups; however, they would have different treatments. For RG2, we would show the culturally resonant advertisements. In other words, within RG2, we further define two subtarget groups: Arab and Filipino groups, as we did in the previous case study. Then, for the Arab group, we show the advertisement with the Arab model, and for the Filipino group, we show the advertisement with the Filipino model. Finally, users in RG3 would be exposed to advertisements that are not culturally resonant. Hence, the Arab group would see the advertisement with the Filipino model, and the Filipino group would see the advertisement with the Arab model.

Similarly, the RCT can be conducted on other social media platforms as long as we are able to define these three RGs. For example, on Snapchat, we can split users using birth year attributes (ie, age). Similar to the birth month attributes, modular operations can be used to split users into 3 RGs. Users born in years in which modulo 3 is equal to 1 (eg, 1984) are RG1 (1 mod 3), users born in years in which modulo 3 is equal to 2 (eg, 1985) are RG2 (2 mod 3), and users born in years in which module 3 is equal to 0 (eg, 1986) are RG0 (0 mod 3). Geographic splitting can be used for Twitter. For example, once all postal codes used for targeting are identified, they can be simply divided into three sets. These random sets of postal codes split users into 3 RGs. Finally, on TikTok, we can conduct an RCT using custom audience. However, this would require a campaign to have a list of known users to draw from.

In accordance with the next step of the framework *Stage 2, Section 1: Splitting the Target Audience Into Smaller Segments*, we define four subtarget groups: (1) RG2 and Arab group, (2) RG2 and Filipino group, (3) RG3 and Arab group, and (4) RG3 and Filipino group. We leave RG1 as it is at this stage as RG1 is the control group, and we would not run the advertisement for that group.

In *Stage 2, Section 2: Assessing the Size of Each Segment by TAT,* we assess the audience size for each subtarget group. For subgroup 1 (RG2 and Arab group), we target Facebook and Instagram users who are women; aged ≥45 years; living in Qatar; speaking Arabic; and born in February, May, August, or November. Similarly, for subgroup 2 (RG2 and Filipino group), we target Facebook and Instagram users who are women; aged 45 ≥years; living in Qatar; used to live in the Philippines; and born in February, May, August, or November. Subgroups 3 and 4 are also defined by almost the same set of attributes, except for the targeted birth months.

In *Stage 3, Section 1*, Tailoring Campaign Content, we assume that we are using the same advertisement content as in the previous case study (Figure 4).

In *Stage 3, Section 2: Testing the Tailored Content for Each Segment*, we run the campaign in a manner similar to that in the previous case study. As our four subgroups are mutually exclusive to each other, we do not use the random A/B testing function provided by Facebook. Instead, we run them as 4 advertisement sets. Once the campaign is complete, the offline data can be analyzed. We note that this analysis does not require any protected health information. However, weekly data aggregated by birth month and nationality or race and ethnicity would be required to evaluate the offline impact.

First, by comparing the number of visits among people belonging to RG1 (born in January, April, etc) with those of RG2 (born in February, May, etc) and RG3 (born in March, June, etc), we can assess whether social media campaigns drive more visits. Then, by comparing the number of visitors whose ethnicity is Arab and who belong to RG2 with those who are Arab and belong to RG3, it is possible to measure the effect of culturally resonant advertisements. Similarly, for the Filipino group, one can compare the number of visitors whose nationality is Filipino and belong to RG2 with those Filipinos who belong to RG3.

Another factor to consider is the time lag between online advertisement exposure and clinic visits. It is not known how long it would take for social media users to visit a clinic once they are exposed to the online advertisement. Measuring the long-term effect of a marketing intervention has been one of the biggest challenges for businesses [43]. Thus, when evaluating the campaign, one may expect to see a time lag of a few days to months.

## Discussion

Inspired by the capability of TATs, we proposed the development of a comprehensive framework to help run public health campaigns using TATs on social media. The PPHC framework aims to support step-by-step guidance and systematic evaluation of the impact of online and offline public health social media campaigns. Our framework considers the overall process of running public health campaigns by estimating the priority audience to evaluate campaigns using various metrics. The PPHC framework relies on two common features of modern TATs on social media: (1) numerical estimation of social media users matching a given set of characteristics and (2) low-cost advertisement delivery. As most social media platforms, including Facebook, Instagram, LinkedIn, Twitter, Snapchat, and TikTok, offer TATs with these 2 features, the PPHC framework we propose is versatile across multiple social media channels. The framework can be used for any number of public health campaigns, as long as the target groups are definable via TATs. It is also flexible enough to evaluate both the online and offline impacts of public health campaigns. Offline metrics have rarely been used in public health campaigns on social media because it is very challenging to establish links between online advertising and the resulting behavior. Certain features of our framework that enable us to conduct RCTs such as split-by-birth month are novel and are important for offline validation.

The concept of PPHCs is not entirely new. Targeted and tailored health content has been used in various health promotion programs to incorporate cultural nuances [44]. For example, culture-centric narrative theory [45] has been shown to be effective in promoting HPV vaccination among college women [46]. It has also been effective in increasing cervical cancer awareness among those of Latina and Mexican American ethnicity [47]. However, our framework expands on these ideas, encouraging tailored approaches via online public health campaigns through TATs beyond offline campaigns.

The social media platform’s large, diverse user base offers a unique opportunity to reach significant chunks of specific populations [21]. A series of studies have already suggested that social media (mainly Facebook) could serve as a channel for health care study recruitment [22-26]. These studies used TATs to leverage target attributes ranging from basic demographics, such as gender and age, to more sophisticated behavioral properties, such as interests. Most of the studies observed that online channels offered a more affordable mechanism to recruit participants than traditional methods (eg, offline surveys). Among existing social media offering TATs, Facebook seems to be the preferred channel in the literature.

In particular, Lane et al [23] reviewed 12 studies on online recruitment methods and determined that Facebook advertisements were the most effective method for implementing targeted advertisements. Moreover, Whitaker et al [48] conducted a comprehensive review of 35 studies that used Facebook as a recruitment tool, reporting that the median value of impressions is 3.3 million and CPC is only US $0.51. In short, it seems that TATs on Facebook can effectively reach the target audience with minimal cost in recruiting participants for health research. Beyond simple demographic-based targeting, some studies have attempted to define a fine-grained target group. For example, Prescott et al [22] used 65 interests to target males aged 14-18 years living in the United States who are adolescent gay, bisexual, and have sex with men. Ultimately, all these previous studies on health communication used targeted, cost-effective TATs to define a specific online target group. However, on the whole, they still ascribe to the *one-size-fits-all* model by exposing all cohort members to the same advertisement.

In contrast, our framework expands on this model, building impact evaluation into the advertisement design process, and target group selection via online and offline behavioral changes. The framework we propose aligns most closely with the approach used by Reiter et al [26]. In their study, Reiter et al [26] evaluated the effects of different images and texts included in social media advertisements to recruit young gay and bisexual men for the pilot test of an online HPV vaccination intervention and found that the text and image in the advertisements are important in advertisement performance. These results corroborate the idea that our framework is effective.

Separately, our framework provides step-by-step guidance to running such experiments using TATs along with a methodology to perform an online RCT to track offline behavior changes, which can better reflect the effectiveness of the campaigns. The framework will help to broaden our understanding of the mechanisms of healthy behavior changes, explaining the factors related to such changes, including users’ psychosocial characteristics and online behavior. Thus, the proposed PPHC framework has the potential to support higher population uptake and engagement rates by encouraging a more standardized, concise, efficient, and targeted approach to public health campaign development. The results of our case study also highlight that advertisement performance can differ in surprising ways across target groups, emphasizing the need for a systematic evaluation of campaign content in advance of campaign launch.

However, it is worth noting that because our framework is based on TATs, the limitations of these tools also naturally become framework limitations.

First, there are concerns about whether social media users are a representative sample of the offline population [49]. However, as Facebook has a very large user base (2.5 billion monthly active users as of December 2019 [50]) and allows advertisers to reach a large audience across age, race, ethnicity, and geographic locations, many researchers have used it to reach users who might have been underrepresented in other forms of sampling.

Second, social media are a powerful communication platform that can be abused, thus requiring careful implementation. Our framework aims to help public health officials optimize the effectiveness of their campaigns. Thus, this should help combat misinformation through the effective dissemination of public health messages. Regarding privacy, although social media platforms contain a lot of individual information, this knowledge is only indirectly made available to advertisers through group targeting rather than individual targeting. Nonetheless, any campaign that targets or personalizes its messages will have to weigh the advantage of specific group targeting with the potential risk of decreasing sample size. There have already been several studies that have addressed the privacy risks of targeted advertisements, particularly when they target very small groups, but such risks have been continuously reported and resolved [51,52].

Third, the campaign may be restricted by the TATs’ policy. For example, Ramo and Prochaska [19] pointed out that the success of their campaign was dependent on Facebook’s approval of the advertisement; one of their advertisements was not approved because of a picture of a marijuana leaf, although they provided evidence of an academic research study. In addition, TATs generally do not support targeting by sensitive information, such as medical history, and no longer allow advertisers to include advertisement content specifying a personal health condition. For example, although Subasinghe et al [53] were able to run the advertisement that targets females aged 18-25 years living in Victoria, Australia, with advertisement text explicitly saying “Are you 18-25 and did NOT receive the cervical cancer vaccine? We need you to help us!” to target unvaccinated women, such verbiage would not be approved at present.

Finally, although our framework largely relies on the features of TATs, domain expertise is still critical for creating and running health campaigns. Input from policymakers and health practitioners is essential for *stages 0* *and 1*. The framework requires domain knowledge of communication experts in *stage 2, section 1* and *stage 3, section 1* to design public health campaigns. Most importantly, a strong partnership with local organizations to run campaigns and collect offline data is essential for ongoing *stages 4* and *5* development. In the future, we hope that our framework integrates efforts from these diverse sectors along with existing TATs to construct a single PPHC workflow.

## Appendix

Multimedia Appendix 1 The full list of traits that targeted advertising tools support.

## Abbreviations

CPC: cost per click
CTR: click-through rate
HPV: human papillomavirus
LGBT: lesbian, gay, bisexual, and transgender
PPHC: precision public health campaign
RCT: randomized controlled trial
RG: random group
TAT: targeted advertising tool
