# Correction: Precision Public Health Campaign: Delivering Persuasive Messages to Relevant Segments Through Targeted Advertisements on Social Media

**DOI:** 10.2196/33922

**Published:** 2021-10-05

**Authors:** Jisun An, Haewoon Kwak, Hanya M Qureshi, Ingmar Weber

**Affiliations:** 1 School of Computing and Information Systems Singapore Management University Singapore Singapore; 2 Yale School of Medicine Yale University New Haven, CT United States; 3 Qatar Computing Research Institute Hamad Bin Khalifa University Doha Qatar

In “Precision Public Health Campaign: Delivering Persuasive Messages to Relevant Segments Through Targeted Advertisements on Social Media” (JMIR Form Res 2021;5(9):e22313) the authors noted one error.

In the originally published manuscript, the order of authors Ingmar Weber and Hanya M Qureshi was reversed. This has been corrected to reflect that Hanya M Qureshi is the paper’s third author and Ingmar Weber is the paper’s fourth author. The author affiliations have been renumbered accordingly.

The full list of authorship and affiliations in the originally published version appeared as follows:

Jisun An^1^, PhD; Haewoon Kwak^1^, PhD; Ingmar Weber^2^, PhD; Hanya M Qureshi^3^, BA

^1^School of Computing and Information Systems, Singapore Management University, Singapore, Singapore

^2^Qatar Computing Research Institute, Hamad Bin Khalifa University, Doha, Qatar

^3^Yale School of Medicine, Yale University, New Haven, CT, United States

The full list of authorship and affiliations has been updated as follows in the corrected version:

Jisun An^1^, PhD; Haewoon Kwak^1^, PhD; Hanya M Qureshi^2^, BA; Ingmar Weber^3^, PhD

^1^School of Computing and Information Systems, Singapore Management University, Singapore, Singapore

^2^Yale School of Medicine, Yale University, New Haven, CT, United States

^3^Qatar Computing Research Institute, Hamad Bin Khalifa University, Doha, Qatar

The correction will appear in the online version of the paper on the JMIR Publications website on October 5, 2021, together with the publication of this correction notice. Because this was made after submission to PubMed, PubMed Central, and other full-text repositories, the corrected article has also been resubmitted to those repositories.

