# Supplementary material for: Precision Public Health Campaign: Delivering Persuasive Messages to Relevant Segments Through Targeted Advertisements on Social Media
Source: JMIR Form Res. 2021 Sep 24;5(9):e22313. doi: 10.2196/22313 (PMC8492044; doi:10.2196/22313)
Supplement: Multimedia Appendix 1 [file formative_v5i9e22313_app1.pdf]

Multimedia Appendix 1. The full list of traits that Targeted Advertising Tools (TATs) support.

1 Facebook/Instagram

1.1 Location Traits

Using Locations, you can target:

- worldwide (e.g., type Worldwide)
- by country group or geographic region (e.g., type in Asia)
- by free trade area (e.g., type in GCC, the Gulf Cooperation Council)
- by sub-regions within a country (e.g., type in Michigan)
- by other features (e.g., type in iTunes app store countries or Emerging markets).

1.2 Demographic Traits

TAT in most of the social media services provides similar capability to target audience by using demographic traits.

- Gender (All, Men, Women)
- Age (13 to 65+)
- Race
- Language
- Education
  - Education Level (At high school, At university, At university (postgraduate), Doctorate degree, Foundation degree, high school leaver, Master's degree, Professional degree, Some high school, Some university, Some university (postgraduate), university graduate, Unspecified)
  - Fields of study

  - Schools/Universities
  - Undergrad years

- Financial / Income
  - Household income: top 5% of ZIP codes (US)
  - Household income: top 10% of ZIP codes (US)
  - Household income: top 10-25% of ZIP codes (US)
  - Household income: top 25-50% of ZIP codes (US)

- Life events
  - Anniversary (within 30 or 31-60 days)
  - Away from family
  - Away from home town
  - Data of birth (Month of birth, Upcoming birthday)
  - Friends of (e.g., friends of close friends of men with a birthday in 0-7 days, friends of people who recently moved)
  - Long-distance relationship
  - New job
  - Newly engaged (3/6/12 months)
  - Newly wed (3/6/12 months)
  - Recently moved

- Parents
  - Parents (All)
  - New parents (0-12 months)
  - Parents (All)
  - Parents with toddlers (aged 1-2)
  - Parents with pre-schooleres (3-5 years)
  - Parents with primary school-age children (aged 6-8 yaers)
  - Parents with preteens (aged 8-12)
  - Parents with adult children (aged 18-26)

- Relationship
  - civil partnership
  - Complicated
  - Divorced

- Domestic partnership
- Engaged
- In a relationship
- Married
- Open relationship
- Separated
- Single
- Unspecified
- Widowed

- Work
  - Employers (e.g., Starbucks)
  - Industries (e.g., Arts, entertainment, sport and media, Business and finance, Farming, fishing and forestry, Healthcare and medical services, Life, physical and social sciences)
  - Job titles

1.3 Behavioral Traits

Different social media services provide different granularity of audience targeting based on behavior traits. For example, Facebook allows to target audience by combining various features, such as where a user lived in before, when a user is celebrating anniversary, or whether a user is frequent traveller, while some of them are not available in Twitter or SnapChat. Below are examples of behavioral traits provided by Facebook:

- Anniversary (within 61-90 days)
- Consumer classification
  - People who prefer high-value goods
  - People who prefer mid- and high-value goods
  - Available for Argentina, Brazil, Chile, India, Indonesia, Kingdom of Saudi Arabia, Malaysia, Mexico, Pakistan, Philippines, South Africa, Turkey, Turkey, UAE
- Digital activities
  - Canvas gaming
  - Console gamers
  - Early technology adopters

- Facebook Payments users (30 days)
- Facebook Payments users (90 days)
- Facebook Payments users (higher than average spend)
- Facebook access: older devices and OS
- Facebook page admins
- Internet browser used
- Operating system used
- Primary email domain
- Small business owners

- Ex-pats
  - Close friends of those who live abroad
  - Family of those who live abroad
  - Lived in a certain country (formerly Ex-pats in that country)
  - Lives abroad
- Mobile Device User
  - All Mobile Devices by Brand (e.g., Amazon, Apple, BlackBerry, Google, HTC, LG, Samsung, Sony)
  - All Mobile Devices by Operating System (e.g., Android, Apple iOD, Windows)
  - Android: 360 degree media not supported
  - Android: 360 degree media supported
  - Facebook access (mobile): all mobile devices
  - Facebook access (mobile): feature phones
  - Facebook access (mobile): smartphones and tablets
  - Facebook access (mobile): tablets
  - Network Connection (2G, 3G, 4G, Wi-Fi)
  - New smartphone and tablet users
  - Owns: OnePlus
- Mobile device user/device use time (e.g., 1-3 months)
- More categories
- Multicultural affinity
  - African American (US)

  - Asian-American (US)
  - Hispanic (US - All)
  - Hispanic (US - Bilingual)
  - Hispanic (US - English dominant)
  - Hispanic (US - Spanish dominant)

- Politics (US)
  - Likely engagement with US political content (conservative)
  - Likely engagement with US political content (liberal)
  - Likely engagement with US political content (moderate)

- Purchase behaviour (e.g., Engaged shoppers)

- Ramadan (Month)
  - Close friends of people celebrating Ramadan
  - Ramadan month (high content engagement)
  - Ramadan month (medium content engagement)

- Soccer
  - Football fans (high content engagement)
  - Football fans (moderate content engagement)
  - Friends of Football fans

- Travel
  - Commuters
  - Frequent international travellers
  - Frequent travellers
  - Returned from travelling one week ago
  - Returned from travelling two weeks ago

1.4 Interests Traits

This interests traits can be mainly divided into nine categories:

- Business and industry (e.g., Advertising, Agriculture, Architecture, Aviation, Banking, Business, Construction, Design, Economics, Engineering, Entrepreneurship, Healthcare, Higher education, Management, Marketing, Nursing)

- Entertainment

  - Films (e.g., Action, Bollywood, Comedy, etc)
  - Games (e.g., First-person shooter games, Massively multiplayer online role-playing games)
  - Live events (e.g., Ballet, Bars, Concerts, Dancehalls, Music festivals, etc)
  - Music (e.g., Blues music, Classical music, Country music, Dance music, Electronic music, Gospel music, etc)
  - Reading (e.g., Books, Comics, Fiction books, Literature, Magazines, etc)
  - Television programme (e.g., TV chat shows, TV comedies, TV game shows, TV reality shows)

- Family and relationships (e.g., Dating, Family, Fatherhood, Friendship, Marriage, Motherhood, Parenting, Weddings)

- Fitness and wellness (e.g., Bodybuilding, Meditation, Physical exercise, Physical fitness, Running, Weight training, Yoga)

- Food and drink
  - Alcoholic drink (e.g., Beer, Distilled drinks, Wine)
  - Cooking (e.g., Baking , Recipes)
  - Cuisine (e.g., Chinese cuisine, French Cuisine, German cuisine, etc)
  - Drinks (e.g., Coffee, Energy drinks, Juice, Soft drinks, Tea)
  - Food (e.g., Barbecue, Chocolate, Desserts, Fast food, Organic food, Pizza, Seafood, Veganism, Vegetarianism)
  - Restaurants (e.g., Coffeehouses, Diners, Fast casual restaurants, Fast food restaurants)

- Hobbies and activities
  - Arts and music (e.g., Acting, Crafts, Dance, etc)
  - Current events
  - Home and garden (e.g., Do it yourself (DIY), Furniture, Gardening, Home appliances)
  - Pets (e.g., Birds, Cats, Dogs, Fish, Horses, etc)
  - Politics and social issues (e.g., Charity and causes, Community issues, Environmentalism, Law, Politics, Religion, etc)
  - Travel (e.g., Holidays, Hotels, Lakes, Mountains, Nature, Theme parks, etc)

  - Vehicles (e.g., 4x4s, Automobiles, Boats, Electric vehicles, Hybrids, etc)

- Shopping and fashion (e.g., Beauty, Clothing, Fashion accessories, Shopping, Toys)
- Sports and outdoors
  - Outdoor recreation (e.g., Boating, Camping, Fishing, Horseback riding, Hunting, Mountain biking, Surfing)
  - Sports (e.g., American football, Baseball, Basketball, Car racing, College football, Football, Golf, Marathons, Skiing, Snowboarding, Swimming, Tennis, Triathlons, Volleyball)
- Technology (e.g., Computers, Consumer electronics)
- Additional interests (e.g., Breast Cancer Awareness, etc)

2 Twitter

2.1 Location Traits

- worldwide
- by country (e.g., type in United States)
- by regions or state (e.g., type in Arizona)
- by metros (e.g., Designated Market Areas (DMAs))
- by cities (e.g., type in Los Angeles)
- by postal codes (e.g., type in 85123)

2.2 Demographic Traits

- Gender (Any, Men, Women)
- All or Age range (13 to 50+)
- Language

2.3 Interests Traits

- Automotive (e.g., Car culture, Luxury, Minivans, etc)
- Beauty (e.g., Body art, Face care, Make-up, Skin care, etc)
- Books and literature (e.g., Biographies and memoirs, Cookbooks, food, and wine, Nonfiction, etc)

- Business (e.g., Advertising, Construction, Green solutions, Nonprofit, etc)
- Careers (e.g., Job fairs, U.S. military, etc)
- Education (e.g., College life, Homeschooling, etc)
- Events (e.g., Entertainment awards, Movie festivals, Political elections, etc)
- Family and parenting (e.g., Babies and toddlers, Elder care, Parenting teens, etc)
- Food and drink (e.g., Barbecues and grilling, Beer, Desserts and baking, Find dining, etc)
- Gaming (e.g., Board gaming, Console gaming, Roleplaying games, etc)
- Health (e.g., Health news and general info)
- Hobbies and interests (e.g., Birdwatching, Dance, Guitar, Scrapbooking, etc)
- Home and garden (e.g., Gardening, Remodeling and construction, etc)
- Law, government, and politics (Conservative, Legal issues, Liberal, Nonpartisan, etc)
- Life stages (e.g., Dads, Moms, Newlyweds, etc)
- Movies and television (e.g., Animation, Bollywood, Documentary, Horror, etc)
- Music and radio (e.g., Blues, Country, DJs, Pop, World, etc)
- Personal finance (e.g., Banking, Hedge funds, Stocks, etc)
- Pets (e.g., Cats, Dogs, Horses, Reptiles, etc)
- Science (e.g., Biology, Geology, Physics, Weather, etc)
- Society (e.g., Dating, Divorce support, Marriage, Senior living, etc)
- Sports (e.g., Boxing, Cycling, Rudby, Sailing, Tennis etc)
- Style and fashion (e.g., Jewelry, Men's shoes, Women's tops, etc)
- Technology and computing (e.g., Antivirus, Databases, MacOS, Web design, etc)
- Travel (e.g., Adventure travel, Camping, Europe, National parks, Traveling with kids, etc)

2.4 Other Traits

Twitter supports the below targeting attributes as well:

- Keywords: Reach people based on keywords in their search queries, recent Tweets, and Tweets they recently engaged with. This includes related terms, stem variations, hashtags, synonyms, misspellings, and slang. Emojis also count.)
- Movies and TV shows: Reach people engaged with specific TV shows and movies, before, during, and after a telecast. The likelihood of someone seeing a movie or TV show is based off their Tweets (in line with when the movie or TV show airs).
- Events: Audiences for event targeting are built based on a number of signals including user Tweet content, behavior, and engagement. (e.g., conferences, holidays, Olympics, etc)
- Conversation topics: Reach people who Tweeted, engaged with a Tweet, or looked at a Tweet about a conversation topic. Conversations have a look-back window of 28 days.

3 Snapchat

3.1 Location Traits

- by country (e.g., type in United States)
- by regions or state (e.g., type in Arizona)
- by metros (e.g., Designated Market Areas (DMAs))
- by postal codes (e.g., type in 85123)

3.2 Demographic Traits

- Gender (All, Men, Women)
- Age (13 to 65+)
- Language
- Education Level
  - Bachelor Degree
  - Graduate Degree
  - High School Diploma
  - Some College

- Some High School

- Financial / Income
  - Household income: $1,000-$24,999
  - Household income: $25,000-$49,999
  - Household income: $50,000-$74,999
  - Household income: $75,000-$99,999
  - Household income: $100,000-$124,999
  - Household income: $125,000-$149,999
  - Household income: $150,000-$174,999
  - Household income: $175,000-$199,999
  - Household income: $200,000-$249,999
  - Household income: $250,000+
- Life events
  - First Time Homebuyer
  - New Homeownder, 12 months
  - New Homeownder, 6 months
  - New Mover, 12 months
  - New Mover, 6 months
  - New Parent, Child 0-3 yrs
  - Recently Married, 12 months
  - Recently Married, 6 months
  - Recently Married, 3 months
  - Recently Single, 12 months
  - Recently Single, 6 months
  - Recently Single, 3 months
- Marital Status
  - Married
  - Single
- Parents
  - Moms (1 child)
  - Moms (2+ children)
  - Moms (Gen X 1965-1979)

  - Moms (Gen Y 1980-1993)
  - Moms (Single)
  - Moms (Married)
  - Moms (of child 0-3 yrs old)
  - Moms (of child 4-6 yrs old)
  - Moms (of child 7-12 yrs old)
  - Moms (of child 13-15 yrs old)
  - Moms (of child 16-18 yrs old)
  - Moms (Stay at Home)
  - Moms (Working)
- Occupation
  - Blue Collar
  - Business, Finance or Management
  - Farming, Fish or Forestry
  - Legal, Education or Health
  - Office & Administrative
  - Retired
  - Sales
  - Tech & Science

3.3 Interests Traits

- Adventure Seekers
- Advocates & Activists
- Arts & Culture Mavens
- Automotive Enthusiasts
- Beachgoers & Surfers
- Beauty Mavens
- Bookworms & Avid Readers
- Chat Fiction Enthusiasts
- Clubbers & Party People
- Collegiates

- Comics & Animation Fans
- Do-It-Yourselfers
- Fashion & Style Gurus
- Film & TV Fans
- Fitness Enthusiasts
- Foodies
- Fun Trivia & Quiz Fanatics
- Gamers
- Green Living Enthusiasts
- High Schoolers
- Hipsters & Trendsetters
- Home Decoristas
- Investors & Entrepreneurs
- Math & Science Enthusiasts
- Meme Watchers
- Men's Lifestyle
- Money Minders
- Music Fans
- New Phone Seekers
- News Watchers
- Outdoor & Nature Enthusiasts
- Parents & Family-Focused
- Pet & Animal Lovers
- Philanthropists
- Photographers
- Shoppers
- Sneakerheads

- Social Drinkers
- Sports Fans
- Techies & Gadget Fans
- Travel Enthusiasts
- Wellness & Healthy Lifestyle
- Women's Lifestyle

4 TikTok

4.1 Location Traits

Using Locations, you can target:

- by country (e.g. type in United States)
- by state (e.g. type in Michigan)

4.2 Demographic Traits

- Gender (All, Men, Women)
- Age range (13-17, 18-24, 25-34, 35-44, 45-54, and 55+)
- Language

4.3 Interests Traits

- Apparel & Accessories
- Appliances
- Apps (e.g., Online shopping, social - messaging, etc)
- Automobiles
- Baby, Kids & Maternity
- Beauty & Personal Care (e.g., Cosmetics, Skin care, etc)
- Education
- Financial Services
- Food & Beverage
- Games

- News & Entertainment (e.g., Anime, Car News, Health & Wellness, Music, etc)
- Pets
- Sports & Outdoors
- Tech & Electronics
- Travel
